# Prognostic nomogram integrating inflammation and nutrition status for acute ischaemic stroke after mechanical thrombectomy

**DOI:** 10.3389/fneur.2026.1647646

**Published:** 2026-02-04

**Authors:** Zhongxiu Wang, Lanqi Li, Chao Li, Mingchao Shi, Dajiang Xing, Shouchun Wang, Chao Wang

**Affiliations:** 1 Center for Rehabilitation Medicine, Department of Neurology, Zhejiang Provincial People's Hospital (Affiliated People's Hospital, Hangzhou Medical College), Hangzhou, China; 2 Department of Neurology, Beihua University, Jilin, China; 3 Stroke Center, Department of Neurology, The First Hospital of Jilin University, Changchun, China

**Keywords:** acute ischaemic stroke, inflammation, mechanical thrombectomy, nutritional status, outcome

## Abstract

**Background:**

The rate of ineffective recanalization after mechanical thrombectomy (MT) is approximately 50% in acute ischaemic stroke patients. Herein, we aim to investigate the correlation of inflammation and nutrition status with clinical outcome and construct a novel prognostic nomogram model to discriminate patients with high risk to facilitate early intervention.

**Methods:**

We conducted a prospective cohort study of a single-center patients with anterior circulation large-artery occlusion who underwent MT between October 2021 and October 2023. Demographic information and clinical characteristics were documented. According to the timing of admission, patients were divided into training and validation cohorts in a 7:3 ratio to developed and verified a prognostic nomogram for 90 day modified Rankin scale score (mRS) 0–3.

**Results:**

Totally, 569 patients were included in the final analysis. The 90 day mRS assessment identified 374 (65.7%) patients with mRS 0–3. Inflammation and nutritional indexes were independent predictors of 90-day mRS 0–3. The areas under the receiver operating curves of the developed nomogram model were 0.865 (95% CI: 0.826–0.905) and 0.861 (95% CI: 0.800–0.922) in the training and validation cohorts, respectively. Decision curve analysis indicated a good net benefit.

**Conclusion:**

Inflammation and nutritional status independently correlated with clinical outcome of anterior circulation large-artery occlusion stroke patients after MT.

## Introduction

Although successful recanalization, defined as an expended treatment in cerebral infarction (eTICI) score of 2b−3, can be achieved in approximately 80% of patients after mechanical thrombectomy, more than 50% of patients can not recover functional independence within 90 days (1). In addition, patients with moderate to large volume of infarction core [ < 100 ml or Alberta stroke programme early CT score (ASPECTS) >3] have shown better outcome than medical treatment, with an acceptable rate of complications after thrombectomy (2–5). In this context, the need for penumbra-based imaging evaluation has been challenged to some extent. Therefore, post-intervention management targeting neuroprotection and complication control might be key factors to further improve prognosis. Infection, particularly, is one of the most common complications after stroke and severely exacerbates brain injury (6, 7). One prior meta-analysis revealed that post-stroke infection accounted for over 48% of the mortality in patients with stroke, whereas the mortality rate in patients with stroke without infections was 18% (8). Moreover, approximately 19%−72% of patients with stroke experience malnutrition, while approximately 30% of patients with stroke experience malnutrition specifically in the recovery phase (9). Malnutrition in patients with stroke results in longer hospital stays and even death (9–12). While 28%−67% of patients with stroke present with swallowing difficulty (13), which is thought to be the cause of malnutrition and aspiration pneumonia (14–16), an increased risk of malnutrition can occur without swallowing difficulties. The effect of inflammation and nutrition status on patients outcome after mechanical thrombectomy has not been fully elucidated. Herein, we constructed a prognosis evaluation model by integrating inflammation and nutrition status to identify high-risk patients so that measures could be taken at an early stage.

## Methods

The study protocol was approved by the hospital ethics committee and conformed to the ethical standards outlined in the Declaration of Helsinki. Informed consent was obtained from either the patients or their legally authorized representatives before study participation.

### Participants

Eligible patients were prospectively and consecutively enrolled from October 2022 to October 2024 in a single-center. Inclusion criteria were as follows: (1) age ≥ 18 years and sex not restricted; (2) disabling symptoms or National Institutes of Health Stroke Scale (NIHSS) ≥ 6 due to acute anterior circulation large vessel occlusion (defined as intracranial internal carotid artery, M1 or M2 segment of middle cerebral artery, A1 segment of anterior cerebral artery, or tandem occlusion) and (3) non-contrast CT or MR diffusion weighted imaging (DWI) ASPECTS ≥ 4, or infarct volume < 100 ml defined by CT perfusion Tmax > 10 s; (4) Initiation of endovascular treatment within 24 h from the last-known-well time; (5) Mechanical thrombectomy was applied as first recanalization method while following intracranial balloon catheter dilation and stenting or thrombolysis as rescuing treatment were acceptable. Exclusion criteria included: (1) evidence of cerebral hemorrhage before intervention; (2) non-disabling symptoms or NIHSS ≤ 5 or neurological deficits due to posterior circulation territory infarction; (3) non-contrast CT or DWI ASPECTS ≤ 3, or infarct volume ≥ 100 ml defined by CT perfusion Tmax > 10 s or early signs of malignant edema or mid-line shift; (4) missing important clinical data, such as follow-up mRS information or critical baseline data. According to the timing of admission, the patients were divided into training and validation cohorts in a 7:3 ratio to construct and verify a prognostic nomogram.

### Endovascular treatment

Endovascular intervention was performed under general anesthesia or sedation. Stent retrievers (Solitaire FR, ev3, Irvine, USA; Trevo ProVue, Stryker Neurovascular, Salt Lake City, USA), aspiration catheters (Penumbra aspiration system, Penumbra Inc., Alameda, USA; React 68/71 catheter, ev3, Irvine, USA), or a combination of both were all acceptable to remove clots. For patients with stenosis or arterial occlusion owing to atherosclerosis, angioplasty with a Gateway (Boston Scientific, Fremont, USA) or Neuro LPS (SinoMed, Tianjin, CHN) intracranial balloon catheter with or without a Wingspan stent (Stryker Neurovascular, Salt Lake City, USA) implantation was permitted as part of the intervention.

### Perioperative management and follow-up

Patients presenting within 4.5 h of symptom onset and without any contraindication received intravenous thrombolysis treatment comprising 0.9 mg/kg recombinant tissue plasminogen activator (rt-PA; Boehringer Ingelheim, Biberach, Germany) after providing informed consent, or were directly moved to the catheter room for endovascular evaluation. Heparinisation by intravenous infusion of unfractionated heparin at 50–100 IU/kg was performed at the start of the procedure, with an additional 1,000 IU administered every hour during the intervention, unless the patient had prior use of intravenous thrombolytic agents or known severe thrombocytopenia and coagulation disorder. Antiplatelet agents, such as tirofiban, were used as necessary. The patients were discharged to their homes or rehabilitation facilities. Postoperative management for all patients included control of underlying risk factors, such as hypertension and diabetes. Additionally, in the absence of any contraindications, all patients were prescribed dual-antiplatelet or anti-coagulation therapy and lipid-lowering drugs, which were maintained for 90 days according to the standard protocol. Ninety-day clinical outcomes were reviewed and collected through face-to-face or telephone interviews by a trained neurologist who were blinded to the quantifying conditions of the patients. To assess potential selection bias, we performed a sensitivity analysis comparing the baseline characteristics of patients who completed follow-up with those lost to follow-up.

### Data collection and definitions

Demographic data and traditional cerebrovascular risk factors were recorded. Stroke severity was scored using NIHSS, while the ASPECTS was used to assess the infarct core. Arterial occlusion location and American Society of Interventional and Therapeutic Neuroradiology/Society of Interventional Radiology (ASITN/SIR) collateral scores were evaluated using digital subtraction angiography (DSA). An eTICI score of 2b−3 was defined as reperfusion success. For the purpose of multivariable modeling, the degree of reperfusion was categorized into eTICI 0–2a, eTICI 2b, and eTICI 2c−3. The mRS score 90 days after endovascular intervention was used to quantify long-term outcomes. The laboratory tests for baseline blood glucose level, blood cell count, C-reactive protein level, and neutrophil-to-lymphocyte ratio were run right before MT. While fasting total cholesterol (TC), triglyceride (TG), and low-density lipoprotein cholesterol (LDL-C) levels were tested in the next morning. Intracranial hemorrhage (ICH) was evaluated within 72 h after MT, symptomatic ICH was defined as new intracranial hemorrhage confirmed by computed tomography or magnetic resonance imaging with an increase in NIHSS score of ≥ 4 points or ≥ 2 points in one category. The nutritional status was assessed by the controlling nutritional status (CONUT) (17) score that use the TC level, lymphocyte count, and serum albumin level according to the following formula: albumin (g/dl): ≥3.5 (0); 3–3.4 (2); 2.5–2.9 (4); < 2.5 (6); total cholesterol (mg/dl): ≥180 (0), 140–179 (1), 100–139 (2), < 100 (3); Lymphocyte count (109/l): ≥1.6 (0), 1.2–1.59 (1), 0.8–1.19 (2), < 0.8 (3). Nutritional status was classified as none (0–1), mild (2–4), moderate (5–8), or severe (9–12) (17). The prognostic nutritional index (PNI) was used as another nutritional scoring system, (18) calculated using the following formula: 10 × serum albumin (g/dl) + 0.005 × total lymphocyte count (/mm^3^). Malnutrition severity was defined as none (>38), moderate (35–38), or severe (< 35) (18).

### Outcome measures

The primary outcome measures were the rate of mRS score of 0–3 during the 90-day follow-up period meaning the capability to ambulate, and the performance of the prognostic nomogram. Secondary outcome measures included recanalization status, symptomatic hemorrhage and all-cause death.

### Statistical analysis

Continuous variables were tested for normality distribution using the Kolmogorov–Smirnov test. Normally distributed variables were presented as the mean ± standard deviation, and means were compared using Student's *t*-test. Non-normally distributed variables were presented as median (interquartile range) or counts and percentages, and were compared using the *U* test. The χ^2^ or Fisher exact tests were used to compare categorical variables. Two-tailed *p* values of < 0.05 were considered statistically significant. Individualized prognostic models for adverse outcomes were constructed using a multivariate logistic regression analysis. Backward elimination based on the Akaike Information Criterion was applied for variable selection. Only participants with complete data on all variables were included in the final multivariable model analysis. A nomogram was subsequently developed and internal validation was conducted by bootstrapping with 1,000 bootstrap samples. Model discrimination was evaluated using the area under the receiver operating characteristic curve (AUROC), and goodness of fit was assessed using the Hosmer–Lemeshow test. Model consistency and accuracy were further examined using calibration curves. Decision curve analysis (DCA) was applied to quantify the net benefit and determine the clinical utility of the nomogram. All tests were two-sided, and statistical significance was defined as *p* < 0.05. All statistical analyses were conducted using the SPSS software version 26 (IBM, New York, NY, USA) and R software (version 4.4.2).

## Results

**Figure 1:**
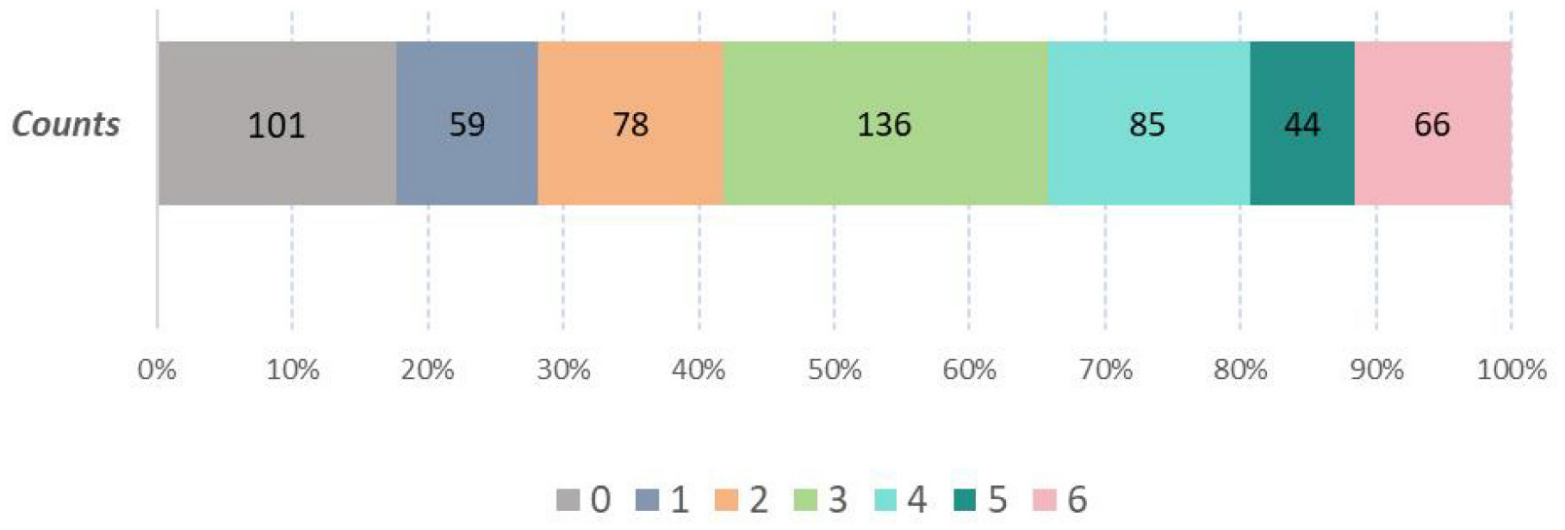
mRS distribution of whole cohort in 90 days.

From October 2021 to October 2023, 623 patients with acute anterior circulation large vessel occlusion received emergency endovascular treatment, of these, 54 patients were lost to follow-up; thus, 569 patients were finally included in the analysis. The distribution of 90-day mRS scores is shown in Figure 1. To quantitatively assess the potential for selection bias, we performed a sensitivity analysis comparing baseline characteristics between patients who completed follow-up and those lost to follow-up. As shown in Supplementary Table S1.

### Baseline characteristics of patients with 90 day mRS 0–3 and 4–6

The 90 day mRS assessment identified 374 (65.7%) patients with mRS 0–3 and 195 (34.3%) patients with mRS 4–6; Univariate analysis showed multiple discrepant characteristics: patients in the mRS 0–3 group had a higher proportion of men, with younger age and lower prevalence of traditional vascular risk factors. Higher ASPECT score and lower NIHSS before and after MT were seen in mRS 0–3 group. Local anesthesia or intravenous sedation, better DSA ASITN/SIR collateral, and shorter puncture-to-recanalization time were related to 90 day mRS 0–3, whereas patients with mRS 4–6 showed higher rate of postoperative hemorrhage, eTICI 0–2a and distal embolizaiton (Table 1).

**Table 1:** Baseline clinical characteristics of patients with 90 day mRS 0–3 and 4–6.

**Characteristics**		**mRS**		***Z*/χ2**	***p* value**
		**mRS 0–3**, ***n*** = **374**	**mRS > 3**, ***n*** = **195**		
Male (*n*, %)		285 (76.2)	113 (57.95)	20.318	0.000^*^
Age (median, Q1, Q3)		61 (51, 69)	66 (58.5, 72.5)	−5.047	0.000^*^
Wake-up stroke (*n*, %)		92 (24.6)	54 (27.69)	0.643	0.423
Hypertension (*n*, %)		214 (57.22)	121 (62.05)	1.236	0.266
Diabetes mellitus (*n*, %)		84 (22.46)	59 (30.26)	4.141	0.042^*^
Baseline NIHSS (median, Q1, Q3)		12 (9, 14)	14 (12, 17.5)	−6.947	0.000^*^
SBP (median, Q1, Q3)		149 (134, 165)	157 (140, 180)	−3.588	0.000^*^
24 h NIHSS after MT (median, Q1, Q3)		8 (4, 11)	14 (11, 22)	−12.101	0.000^*^
Serum glucose (median, Q1, Q3)		7.1 (6.07, 8.45)	7.93 (6.63, 9.6)	−3.887	0.000^*^
White blood cell (median, Q1, Q3)		8.96 (6.98, 11.07)	9.70 (7.72, 12.28)	−3.292	0.001^*^
Neutrophil (median, Q1, Q3)		6.54 (4.84, 8.82)	7.84 (5.64, 10.24)	−3.858	0.000^*^
Neutrophil ratio (median, Q1, Q3)		0.75 (0.68, 0.82)	0.80 (0.73, 0.85)	−4.352	0.000^*^
Lymphocyte (median, Q1, Q3)		1.445 (1.05, 1.89)	1.27 (0.9, 1.66)	−3.391	0.001^*^
Neutrophil/Lymphocyte (median, Q1, Q3)		4.43 (2.96, 6.86)	6.26 (3.88, 9.27)	−4.718	0.000^*^
Serum albumin (median, Q1, Q3)		36.6 (34.5, 38.8)	35.4 (32.9, 38.4)	−3.233	0.001^*^
C-reactive protein (median, Q1, Q3)		7.16 (3.04, 18.53)	16.03 (4.85, 40.53)	−5.073	0.000^*^
TG (median, Q1, Q3)		1.59 (1.08, 2.23)	1.59 (1.2, 2.42)	−0.793	0.428
LDL (median, Q1, Q3)		2.76 (2.24, 3.25)	2.65 (2.11, 3.25)	−1.124	0.261
Total cholesterol (median, Q1, Q3)		4.46 (3.8, 5.08)	4.30 (3.59, 5.09)	−0.903	0.367
CONUT (median, Q1, Q3)		2 (1, 4)	3 (2, 5)	−5.362	0.000^*^
PNI (median, Q1, Q3)		44.4 (41.25, 47)	42.5 (38.63, 45.31)	−5.064	0.000^*^
CONUT: absent (*n*, %)		115 (33.4)	33 (18.8)	26.784	0.000^*^
CONUT: mild (*n*, %)		187 (54.4)	96 (54.5)		
CONUT: moderate (*n*, %)		41 (11.9)	41 (23.3)		
CONUT: severe (*n*, %)		1 (0.3)	6 (3.4)		
PNI: absent (*n*, %)		330 (91.9)	152 (80.4)	16.281	0.000^*^
PNI: moderate (*n*, %)		18 (5.0)	19 (10.1)		
PNI: severe (*n*, %)		11 (3.1)	18 (9.5)		
Coronary heart disease (*n*, %)		41 (10.96)	44 (22.56)	13.576	0.000^*^
Atrial fibrillation (*n*, %)		60 (16.04)	49 (25.13)	6.832	0.009^*^
Premorbid mRS score (*n*, %)	0	342 (91.4)	171 (87.7)		0.362
	1	20 (5.3)	15 (7.7)		
	2	12 (3.2)	9 (4.6)		
Prior ischemic stroke/TIA (*n*, %)		91 (24.33)	78 (40.00)	15.07	0.000^*^
Smoking (*n*, %)		212 (56.68)	76 (38.97)	16.083	0.000^*^
Drinking (*n*, %)		200 (53.48)	67 (34.36)	18.808	0.000^*^
ASPECTS baseline (median, Q1, Q3)		9 (8, 10)	9 (7, 10)	−3.266	0.001^*^
Intravenous thrombolysis (*n*, %)		41 (10.96)	25 (12.82)	0.431	0.511
Anesthesia (*n*, %)	Common	234 (62.57)	164 (84.1)	28.54	0.000^*^
	Sedation	33 (8.82)	9 (4.62)		
	Local	107 (28.61)	22 (11.28)		
Tandem lesion(median, Q1, Q3)		81 (21.66)	59 (30.26)	5.109	0.024^*^
TOAST (*n*, %)	Large artery atherosclerosis	187 (50.00)	106 (54.36)	3.742	0.442
	Cardiac embolism	86 (23.00)	48 (24.61)		
	Other determined etiology	23 (6.15)	12 (6.15)		
	Undetermined etiology	78 (20.86)	29 (14.87)
IIbIIIa receptor inhibitor (*n*, %)		182 (48.66)	84 (43.08)	1.607	0.205
ASITN/SIR (*n*, %)	0–1	152 (40.64)	96 (49.23)	8.234	0.041^*^
	2	136 (36.36)	71 (36.41)		
	3–4	76 (20.32)	27 (13.85)		
Onset to groin puncture time (median, Q1, Q3)		379.5 (254.5, 693.75)	393 (239, 652)	−0.362	0.717
Puncture to reperfusion time (median, Q1, Q3)		69 (50, 100)	90 (69, 129)	−5.669	0.000^*^
eTICI 0–2a (*n*, %)		38 (10.16)	59 (30.26)	52.529	0.000^*^
72 h ICH (*n*, %)		91 (24.33)	78 (40)	40.896	0.000^*^
72 h sICH (*n*, %)		0 (0)	20 (10.26)	41.82	0.000^*^
Distal Embolization (*n*, %)		53 (14.17)	47 (24.1)	8.727	0.003^*^
Discharge destinations (*n*, %)	Home	131 (35.0)	62 (31.8)	1.81	0.613
	Inpatient Rehabilitation facility	94 (25.1)	58 (39.7)		
	Skilled nursing facility	112 (30.0)	59 (30.2)		
	Other/Transfer	37 (9.9)	16 (8.2)		
Complete 90 day anti-platelet/coagulation therapy (*n*, %)		321 (85.8)	160 (82.1)	1.399	0.237

Data are mean ± standard deviation, median (interquartile range), or frequency (percent). “ASITN/SIR, American Society of Intervention Therapeutic Neuroradiology/Society of Interventional Radiology; ASPECTS, Alberta stroke program early CT score; CONUT, controlling nutritional status score; ICH, intracranial hemorrhage; LDL, low density lipoprotein cholesterol; NIHSS, National Institutes of Health Stroke Scale; eTICI, expended thrombolysis in cerebral infarction; PNI, prognostic nutritional index; TG, triglyceride; SBP, systolic blood pressure; sICH, symptomatic intracranial hemorrhage; TOAST, trial of org 10172 in acute stroke treatment; WBC, white blood cell.

### Inflammation and nutritional status

Significant differences were found in inflammation and nutritional indexes between 90 day mRS 0–3 and 4–6 groups (Table 1); Patients with 90 day mRS 4–6 showed higher white blood cell counts, neutrophil counts, neutrophil/lymphocyte ratios, and C-reactive protein levels, while lymphocyte count and albumin were lower (*p* < 0.05). Patients with a CONUT evaluation of malnutrition as “none” had a higher probability of achieving an mRS score of 0–3 compared with patients with malnutrition levels of “mild”, “moderate”, or “severe”, which is consistent with the results found using the PNI scoring system. These differences indicate that patients with severe inflammation and malnutrition stress are more likely to be disabled after MT.

### Analysis of prognostic factors

Multivariate logistic regression analysis identified female sex, baseline NIHSS score, C-reactive protein, puncture to recanalization time, history of cerebrovascular disease, ICH after MT and insufficient re-perfusion (eTICI 0–2a) as adverse factors for mRS 0–3 (OR < 1, *p* < 0.05); Compared with “moderate to severe” malnutrition patients based on the CONUT criteria, patients with “none” or “mild” malnutrition had a higher chance of achieving mRS 0–3 (none: OR: 4.813 (2.119–10.935), *p* < 0.001, mild: OR: 2.651 (1.340–5.242), *p* = 0.002) (Table 2). Table 3 showed efficacy of prognostic models based on multivariate logistic regression analysis. Supplementary Figure S1 showed that CRP and malnutrition status contributes to better performance of the model: AUC: 0.845 (0.812–0.877) vs. 0.808 (0.773, 0.845), *p* = 0.0002.

**Table 2:** Multivariate logistic regression analysis for 90 day mRS 0–3.

**Variables**	***p* value**	**OR**	**95% CI**	
			**Lower**	**Upper**
Age	0.235	0.985	0.96	1.01
Female	0.014^*^	0.423	0.213	0.838
Smoking	0.937	1.028	0.519	2.038
Drinking	0.482	1.287	0.637	2.602
Baseline NIHSS	0.000^*^	0.871	0.816	0.931
Hypertension	0.683	1.121	0.648	1.939
Diabetes mellitus	0.527	0.822	0.448	1.508
Coronary heart disease	0.132	0.578	0.284	1.179
Atrial fibrillation	0.909	1.04	0.529	2.047
Prior ischemic stroke/TIA	0.003^*^	0.409	0.226	0.737
Intravenous thrombolysis	0.361	0.709	0.339	1.484
C-reactive protein	0.000^*^	0.986	0.979	0.994
Onset to groin puncture time	0.262	0.993	0.982	1.005
Puncture to reperfusion time	0.001^*^	0.99	0.983	0.996
ASPECTS baseline	0.177	1.112	0.953	1.296
eTICI: 2c−3	0.000^*^	Reference		
eTICI: 0–2a	0.000^*^	0.117	0.058	0.238
eTICI: 2b	0.002^*^	0.381	0.205	0.706
72h ICH	0.002^*^	0.409	0.233	0.719
CONUT: moderate-severe	0.001^*^	Reference		
CONUT: absent	0.000^*^	4.813	2.119	10.935
CONUT: mild	0.005^*^	2.651	1.34	5.242

“^*^” indicates *p* < 0.05; CI, confidence interval; CONUT, controlling nutritional status score; eTICI, expended thrombolysis in cerebral infarction; NIHSS, National Institutes of Health Stroke Scale; OR, odds ratio; TIA, transient ischemic attack.

**Table 3:** Comparison of the efficacy of prognostic models with or without CRP and CONUT.

**Prognostic models**	**AUC**	**AUC 95% CI**	***p* value for Delong test**
Model 1	0.845	0.812–0.877	0.0002^*^
Model 2	0.808	0.773–0.845	

Model 1 was based on logistic regression analysis and included all the significant variables: NIHSS + CRP + Puncture to reperfusion time + gender + Prior ischemic stroke/TIA + eTICI+ ICH72H + CONUT; Model 2 included all the variants in Model 1 except CRP and CONUT. AUC, area under the curve; CI, confidence interval; CONUT, controlling nutritional status score; CRP, C reactive protein.

*p* value for Delong test “^*^” indicates *p* < 0.05.

### Construction and verification of prognostic model

The overall population was divided into the training (*n* = 398) and validation (*n* = 171) cohorts in a 7:3 ratio according to the chronological order of enrollment. Characteristics of the two cohorts were shown in Table 4. The two cohorts were well-balanced, with no statistically significant differences observed in demographic features, stroke severity, laboratory parameters (including inflammation and nutrition markers), comorbidities, or treatment modalities (all *p* > 0.05).

**Table 4:** Comparison of clinical characteristics of training and validation cohort.

**Characteristics**		**Training cohort *n* = 398**	**Validation cohort *n* = 171**	***Z*/χ2**	***p* value**
Male (*n*, %)		281 (70.6)	117 (68.4)	1.46	0.227
Age (median, Q1, Q3)		63.5 (54, 69)	63 (53, 70)	−0.037	0.970
Wake-up stroke (*n*, %)		109 (27.4)	37 (21.6)	2.119	0.146
Hypertension (*n*, %)		229 (57.5)	106 (62)	0.979	0.323
Diabetes mellitus (*n*, %)		99 (24.9)	44 (25.7)	0.047	0.829
Baseline NIHSS (median, Q1, Q3)		13 (9.5, 16)	12 (10, 15)	−0.134	0.893
SBP (median, Q1, Q3)		150 (135, 170)	152 (137, 170)	−0.712	0.476
24 h NIHSS after MT (median, Q1, Q3)		10 (6, 14)	10 (5, 13)	−1.126	0.260
Serum glucose (median, Q1, Q3)		7.40 (6.20, 9.01)	7.20 (6.14, 8.70)	−0.876	0.381
White blood cell (median, Q1, Q3)		9.16 (7.26, 11.60)	9.39 (6.92, 11.59)	−0.065	0.948
Neutrophil (median, Q1, Q3)		6.96 (5.04, 9.14)	7.08 (5.03, 9.38)	−0.195	0.845
Neutrophil ratio (median, Q1, Q3)		0.76 (0.69, 0.84)	0.78 (0.71, 0.84)	−0.385	0.700
Lymphocyte (median, Q1, Q3)		1.41 (1.01, 1.84)	1.32 (0.98, 1.68)	−1.126	0.260
Neutrophil/Lymphocyte (median, Q1, Q3)		4.79 (3.04, 7.79)	5.22 (3.42, 7.78)	−0.904	0.366
Serum albumin (median, Q1, Q3)		36.25 (33.90, 38.70)	36.15 (33.48, 38.40)	−0.62	0.535
C-reactive protein (median, Q1, Q3)		8.97 (3.49, 29.53)	8.48 (4.00, 22.21)	−0.667	0.505
TG (median, Q1, Q3)		1.63 (1.13, 2.35)	1.50 (1.09, 2.08)	−1.181	0.238
LDL (median, Q1, Q3)		2.65 (2.12, 3.25)	2.81 (2.37, 3.16)	−1.642	0.101
Total cholesterol (median, Q1, Q3)		4.32 (3.71, 5.10)	4.49 (3.82, 5.04)	−0.613	0.540
CONUT (median, Q1, Q3)		3 (1, 4)	3.00 (1, 4)	−0.593	0.553
PNI (median, Q1, Q3)		44.05 (40.50, 46.53)	43.35 (39.85, 46.00)	−1.385	0.166
CONUT: absent (*n*, %)		110 (29.6)	38 (25.7)		0.555
CONUT: mild (*n*, %)		203 (54.6)	80 (54.1)
CONUT: moderate (*n*, %)		55 (14.8)	27 (18.2)
CONUT: severe (*n*, %)		4 (1.1)	3 (2.0)
PNI: absent (*n*, %)		348 (90.0)	134 (83.2)	5.19	0.075
PNI: moderate (*n*, %)		23 (5.9)	14 (8.7)
PNI: severe (*n*, %)		16 (41)	13 (8.1)
Coronary heart disease (*n*, %)		64 (16.1)	21 (12.3)	1.359	0.244
Atrial fibrillation (*n*, %)		78 (19.6)	31 (18.1)	0.167	0.683
Premorbid mRS score (*n*, %)	0	360 (90.5)	153 (89.5)	1.604	0.448
	1	22 (5.5)	13 (7.6)		
	2	16 (4)	5 (2.9)		
Prior ischemic stroke/TIA (*n*, %)		111 (27.9)	58 (33.9)	2.082	0.149
Smoking (*n*, %)		201 (50.5)	84 (49.1)	0.218	0.641
Drinking (*n*, %)		186 (46.7)	81 (47.4)	0.019	0.889
ASPECTS baseline (median, Q1, Q3)		9 (8, 10)	9 (8, 10)	−0.314	0.754
Intravenous thrombolysis (*n*, %)		39 (9.8)	27 (15.8)	0.019	0.889
Anesthesia (*n*, %)	Common	286 (71.9)	112 (65.5)	5.355	0.069
	Sedation	23 (5.8)	19 (11.1)		
	Local	89 (22.4)	40 (23.4)		
Tandem lesion (median, Q1, Q3)		89 (22.4)	51 (29.8)	3.591	0.058
TOAST (*n*, %)	Large artery atherosclerosis	205 (51.5)	88 (51.5)	0.071	0.995
	Cardiac embolism	94 (23.6)	40 (23.4)		
	Other determined etiology	25 (6.3)	10 (5.8)		
	Undetermined etiology	74 (18.6)	33 (19.3)		
IIbIIIa receptor inhibitor (*n*, %)		190 (47.7)	76 (44.4)	0.521	0.470
ASITN/SIR (*n*, %)	0–1	75 (18.8)	28 (16.4)	1.769	0.413
	2	151 (37.9)	56 (32.7)		
	3–4	168 (42.2)	80 (46.8)		
Onset to groin puncture time, hour (median, Q1, Q3)		6.38 (4.00, 11.24)	6.25 (4.50, 11.85)	−0.645	0.519
Puncture to reperfusion time, minute (median, Q1, Q3)		75 (55, 104)	84 (52, 114.5)	−1.065	0.287
eTICI 0–2a (*n*, %)		73 (18.3)	24 (14.0)	2.698	0.260
72 h ICH (*n*, %)		120 (30.2)	49 (28.7)	0.128	0.720
72 h sICH (*n*, %)		14 (3.5)	6 (3.5)	0	0.994
Distal embolization (*n*, %)		68 (17.1)	32 (18.7)	0.219	0.640
Discharge destinations (*n*, %)	Home	135 (33.9)	58 (33.9)	0.008	1.000
	Inpatient rehabilitation facility	106 (26.6)	46 (26.9)		
	Skilled nursing facility	120 (30.2)	51 (29.8)		
	Other/Transfer	37 (9.3)	16 (9.4)		
Complete 90 day anti-platelet/coagulation therapy (*n*, %)		335 (84.2)	146 (85.4)	0.134	0.715

Data are mean ± standard deviation, median (interquartile range), or frequency (percent). ASITN/SIR, American Society of Intervention Therapeutic Neuroradiology/Society of Interventional Radiology; ASPECTS, alberta stroke program early CT score; CONUT, controlling nutritional status score; ICH, intracranial hemorrhage; LDL, low density lipoprotein cholesterol; NIHSS, National Institutes of Health Stroke Scale; eTICI, expended thrombolysis in cerebral infarction; PNI, prognostic nutritional index; TG, triglyceride; SBP, systolic blood pressure; sICH, symptomatic intracranial hemorrhage; TOAST, trial of org 10172 in acute stroke treatment; WBC, white blood cell.

**Figure 2:**
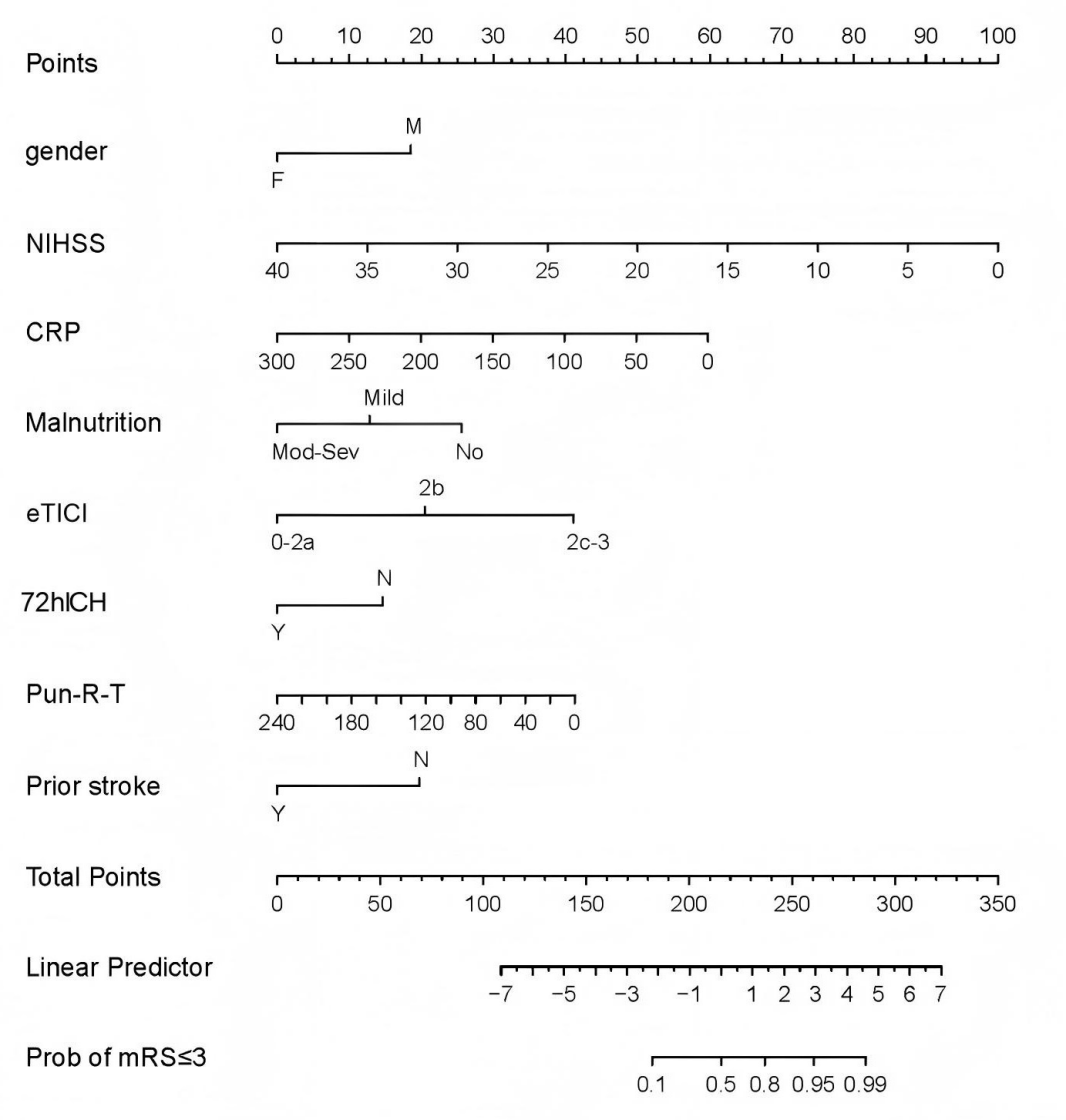
Nomogram for 90 day mRS ≤ 3 after mechanical thrombectomy. This nomogram comprises two areas, in which the upper section was used to assign a score to each variable by projecting its value onto a score line. The lower section was used to calculated the probability (Prob) of patients recovering to mRS ≤ 3 in 90 days after summing all the scores and project the total value onto the scale line of “Prob of mRS ≤ 3”. CRP, C reactive protein; ICH, intracranial hemorrhage; eTICI, expended thrombolysis in cerebral infarction; NIHSS, National Institutes of Health Stroke Scale; Pun-R-T, puncture to reperfusion time.

Significant variables identified using multivariate logistic regression were applied in the training cohort to construct a nomogram (Figure 2) which was then tested for effectiveness in the validation cohort.

**Figure 3:**
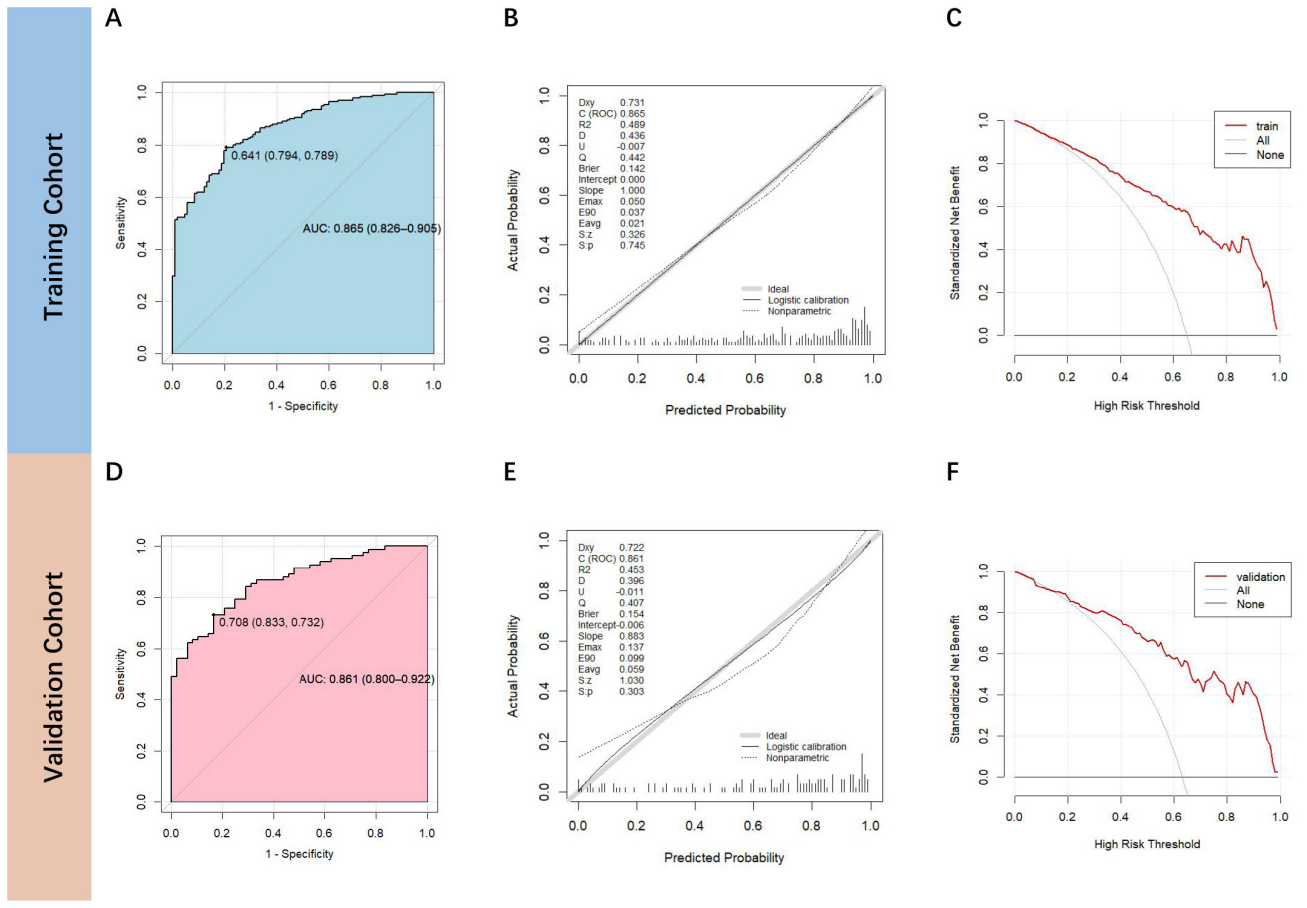
AUROC, calibration and decision curve analysis of the nomogram predictive model. Receiver operating characteristic (ROC) curve was used to illustrates the performance of the model. The area under the ROC represented the sensitivity of the model for predicting 3-month outcomes in training cohort **(A)** and validation cohort **(D)**. Calibration curve depicted the calibration of the model in terms of the agreement between the predicted probability and the observed outcome. The *x*-axis showed the predicted probability using the nomogram, and the *y*-axis showed the actual rate of outcome. The nomogram model showed good calibration ability both in training **(B)** and validation cohorts **(E)**. Decision curve analysis measured the net benefit for each possible threshold probability in training cohort **(C)** and validation cohort **(F)**.

The AUROC values of this nomogram in the training and validation cohorts were 0.865 (95% CI: 0.826–0.905) (Figure 3A) and 0.861 (95% CI: 0.800–0.922) (Figure 3D), respectively, indicating that the model had good discrimination. The calibration curve indicated that the model had reliable predictive accuracy. The *p* values for Spiegelhalter's *Z*-test were 0.745 and 0.303, respectively, indicating no significant departure from a perfect fit. The Brier values were 0.142 and 0.154, indicating that the calibration curves were close to the ideal curve (Figures 3B, E). Also, the DCA curve indicated a relatively favorable net clinical benefit (Figures 3C, F).

## Discussion

In our study, 374 (65.7%) out of 569 patients achieved mRS 0–3 within 90 days after MT treatment for occlusion of the intracranial artery in the anterior circulation. We found that inflammation and nutritional status are independent prognostic factors after MT. C-reactive-protein and white blood cell/neutrophil ratios can reflect the patient's inflammatory stress or infectious complications and significantly correlate with the patient's prognosis. Also, patients with a CONUT evaluation of malnutrition as “none” had a higher probability of achieving an mRS score of 0–3 compared with patients with malnutrition levels of “mild”, “moderate”, or “severe”. These differences indicate that patients with severe inflammation and malnutrition stress are more likely to experience ineffective after MT. Consistent with previous literature, our study found that higher baseline NIHSS, longer puncture to recanalization time, history of cerebrovascular disease and ICH within 72 h after intervention, older age, lower ASPECTS and worse collateral circulation and general anesthesia were associated with worse outcome. Although mechanical thrombectomy could reach over 80% of successful recanalization defined as eTICI 2b−3, the occurrence of ineffective reperfusion is high even in patients with a radiologically-define “small infarct core and large penumbra” before intervention. By integrating these clinical indicators, researchers have focused on developing prognostic scoring tools or prediction models for patients undergoing reperfusion therapy, such as the DRAGON score (19, 20), Houston Intra-Arterial Therapy 2 (HIAT2) score (21), and The Totaled Health Risks in Vascular Events (THRIVE) score (22). External validation studies of these tools demonstrated varied predictive performance. In a systematic review and external validation study (23), Kremers et al. evaluated 19 prediction models for functional outcome after endovascular thrombectomy (EVT) in patients with acute ischemic stroke due to anterior circulation large vessel occlusion. Using data from the MR CLEAN registry (*n* = 3,156), they found that discriminative performance, measured by the area under the curve (AUC), varied widely from 0.61 [SPAN-100 (24)] to 0.80 [MR PREDICTS (25)]. This suggests that prognostic models developed from studies with substantial heterogeneity in population characteristics and observation timepoints often face challenges in their generalizability across broader clinical settings. Beyond well-established conventional factors such as symptom scores, radiological biomarkers, our findings indicate that inflammatory and nutritional indicators are also significant factors influencing patient prognosis. We found that within 72 h after MT, the performance of the nomogram with the inclusion of C-reactive protein and the CONUT score achieved AUROC = 0.865 (95% CI: 0.826–0.905) and 0.861 (95% CI: 0.800–0.922) in the training and validation cohorts respectively which indicated good discrimination, and decision curve analysis also indicated a good net benefit. We believe that perioperative intensive care and preoperative evaluation hold comparable clinical significance in optimizing patient outcomes.

Inflammation-mediated tissue damage is caused by adverse factors and was involved in the entire process in ischemic brain injury (26). Simultaneously, acute brain injury causes an increase in catecholamine and cortisol levels, which induces the apoptosis and inactivation of peripheral lymphocytes (27) and impairs the host's defense against pathogens. Infection is one of the most common complications after stroke and severely exacerbates brain injury (6, 7). Inflammatory cytokines are released from the damaged brain tissue after ischaemia; consequently, the microglia are activated and circulating immune cells are recruited (28). During the acute phase, the influx of polymorphonuclear cells and monocytes is one of the earliest events in the inflammatory cascade. Inflammatory cytokines are cytotoxic and cause increased capillary permeability, thrombosis, and brain oedema progression (29, 30). The aggregation of white blood cells and platelets occurs after endothelial injury, leading to microvascular blockage which affects tissue perfusion at the capillary level. Therefore, even if large blood vessels are successfully reopened, the brain tissue cannot be effectively reperfused and inevitably become infarction. Animal studies have identified the phenomenon of “no reflow” mediated by white blood cells and platelets (31, 32). In addition, specific regulatory lymphocyte subsets participate in the repair process by inhibiting the production of proinflammatory cytokines and regulating microglial activity, thereby maintaining immune homeostasis and exerting immunoregulatory effects (33–35).

The association between inflammatory-nutritional status and functional outcome observed in our study can be explained by several intertwined biological pathways. Firstly, a systemic inflammatory state is known to promote microvascular injury and disrupt the blood-brain barrier, exacerbating cerebral edema and impairing perfusion in the ischemic penumbra. Secondly, both inflammation and malnutrition create a catabolic milieu that is detrimental to neuronal repair and synaptic plasticity in the critical post-stroke recovery phase. Specifically, elevated inflammatory cytokines can directly inhibit neurogenesis and synaptogenesis, while inadequate nutritional support deprives the brain of essential substrates required for energy production and axonal remodeling. There have been reports concerning the impact of inflammation and nutritional status on the prognosis of patients with stroke. However, only a few patients were enrolled following MT. In this context, we constructed this prognostic nomogram through an integrated analysis of a large number of clinical variables, which achieved good discrimination and calibration and excellent performance through external validation. At the same time, the clinical decision curve of this model also showed good net benefits, indicating that it has good clinical practical value. One prior meta-analysis revealed that post-stroke infection accounted for over 48% of the mortality in patients with stroke, whereas the mortality rate in patients with stroke without infections was 18% (8). Moreover, approximately 19%−72% of patients with stroke experience malnutrition, while approximately 30% of patients with stroke experience malnutrition specifically in the recovery phase (9). Malnutrition in patients with stroke results in longer hospital stays and even death (9–12). While 28%−67% of patients with stroke present with swallowing difficulty (13), which is thought to be the cause of malnutrition and aspiration pneumonia (14–16), an increased risk of malnutrition can occur without swallowing difficulties. Consequently, appropriate nutritional supplementation may have a beneficial effect in patients.

Our study has some limitations: First, this is an observational study of a limited group of patients and 54 patients (9%) were lost to follow-up, that might cause selective bias and compromise the robustness of the results, therefore limiting the generalizability of the findings to other populations and clinical settings, the generalizability of our nomogram requires external validation in broader, multi-center cohorts. Second, there is a lack of specific cause-effect analysis for the increase in inflammatory indicators. It is therefore necessary to further distinguish between infectious and non-infectious factors, and assess the impact of antibiotic or anti-inflammatory therapy on patient prognosis. Third, the occurrence rates of serious adverse events, such as large core cerebral infarction, malignant cerebral oedema, and pneumonia, which were also highly sensitive to outcome and might have influenced the results of this study. Finally, the relatively short-term (3-month) follow-up limits our understanding of the long-term prognostic impact of these biomarkers. Subsequent, better designed interventional studies are required to further investigate these topics.

## Conclusion

Inflammation and nutritional status are independent outcome factors following MT in patients with acute ischaemic stroke. A prognostic nomogram based on integrated clinical indicators showed good performance and clinical application.

## Data Availability

The raw data supporting the conclusions of this article will be made available by the authors, without undue reservation.
