# Supplementary material for: Prognostic nomogram integrating inflammation and nutrition status for acute ischaemic stroke after mechanical thrombectomy
Source: Front Neurol. 2026 Feb 4;17:1647646. doi: 10.3389/fneur.2026.1647646 (PMC12913149; doi:10.3389/fneur.2026.1647646)
Supplement: Supplementary file 1 [file Table_1.docx]

**Table S1 Baseline characteristics between patients completed follow-up and those lost to follow-up**

| **Characteristics** | | **Group** | | **Z/χ2** | **p value** |
| --- | --- | --- | --- | --- | --- |
|  |  | **completed follow-up,** n=569 | **lost to follow-up,**n=54 |  |  |
| Male (n, %) | | 398 (69.9%) | 33 (61.1%) | -1.16 | 0.179 |
| Age (median, Q1, Q3) | | 63.00 (54.00,70.00) | 60.00 (50.00,69.75) | -1.63 | 0.455 |
| Hypertension (n, %) | | 335 (58.9%) | 30 (55.6%) | -1.53 | 0.636 |
| Diabetes mellitus (n, %) | | 143 (25.1%) | 14 (25.9%) | -0.24 | 0.898 |
| Baseline NIHSS | | 13.00 (10.00,16.00) | 14.50 (11.00,17.00) | -0.49 | 0.088 |
| SBP (median, Q1, Q3) | | 151.00 (135.00,170.00) | 150.50 (130.25,169.75) | -0.79 | 0.507 |
| Serum glucose (median, Q1, Q3) | | 7.40 (6.20,8.95) | 7.20 (6.38,8.79) | -1.26 | 0.715 |
| White blood cell (median, Q1, Q3) | | 9.27 (7.20,11.61) | 8.87 (6.50,10.69) | -0.3 | 0.192 |
| Lymphocyte (median, Q1, Q3) | | 1.38 (1.00,1.82) | 1.44 (1.20,1.89) | 5.82 | 0.245 |
| Serum Albumin (median, Q1, Q3) | | 36.20 (33.80,38.60) | 36.90 (34.73,40.05) | -1.16 | 0.102 |
| C-reactive protein (median, Q1, Q3) | | 8.86 (3.57,24.75) | 5.38 (3.02,16.08) | -1.63 | 0.125 |
| TG (median, Q1, Q3) | | 1.59 (1.11,2.29) | 1.67 (1.14,2.35) | -1.53 | 0.811 |
| LDL (median, Q1, Q3) | | 2.72 (2.20,3.25) | 2.76 (2.24,3.28) | -0.24 | 0.622 |
| Total cholesterol (median, Q1, Q3) | | 4.37 (3.74,5.08) | 4.65 (3.83,5.27) | -0.49 | 0.432 |
| CONUT (median, Q1, Q3) | | 3.00 (1.00,4.00) | 2.00 (1.00,4.00) | -0.79 | 0.206 |
| PNI (median, Q1, Q3) | | 43.68 (40.34,46.45) | 44.23 (40.66,47.00) | -1.26 | 0.766 |
| CONUT: absent (n, %) | | 148 (28.5%) | 16 (30.2%) | -0.3 | 0.121 |
| CONUT: mild (n, %) | | 283 (54.4%） | 28 (52.8%) |  |  |
| CONUT: moderate (n, %) | | 82 (15.8%) | 6 (11.3%) |  |  |
| CONUT: severe (n, %) | | 7 (1.3%) | 3 (5.7%) |  |  |
| PNI: absent (n, %) | | 482 (88.0%) | 49 (90.7%) | 5.06 | 0.08 |
| PNI: moderate (n, %) | | 37 (6.8%) | 0 (0.0%) |  |  |
| PNI: severe (n, %) | | 29 (5.3%) | 5 (9.3%) |  |  |
| coronary heart disease (n, %) | | 85 (14.9%) | 10 (18.5%) | 0.49 | 0.484 |
| Atrial fibrillation (n, %) | | 109 (19.2%) | 9 (16.7%) | 0.2 | 0.655 |
| Premorbid mRS score (n,%) | 0 | 513(90.2%) | 48（88.9%） | 0.13 | 0.936 |
|  | 1 | 35(6.2%) | 4（7.4%） |  |  |
|  | 2 | 21(3.6) | 2（3.7%） |  |  |
| Prior ischemic stroke/ TIA (n, %) | | 169 (29.7%) | 16 (29.6%) | 0 | 0.991 |
| Smoking (n, %) | | 288 (50.6%) | 27 (50.0%) | 0.01 | 0.931 |
| Drinking (n, %) | | 267 (46.9%) | 19 (35.2%) | 2.74 | 0.098 |
| ASPECTS baseline (median, Q1, Q3) | | 9.00 (8.00,10.00) | 9.00 (8.00,10.00) | 1.19 | 0.236 |
| Intravenous thrombolysis (n, %) | | 66 (11.6%) | 7 (13.0%) | 0.09 | 0.766 |
| Anesthesia (n, %) | common | 398 (69.9%) | 35 (64.8%) | 0.65 | 0.724 |
|  | sedation | 42 (7.4%) | 5 (9.3%) |  |  |
|  | local | 129 (22.7%) | 14 (25.9%) |  |  |
| Tandem lesion(n, %) | | 140 (24.6%) | 8 (14.8%) | 2.61 | 0.106 |
| TOAST (n, %) | Large artery atherosclerosis | 293 (51.5%) | 31 (57.4%) | 4.88 | 0.3 |
|  | Cardiac embolism | 122 (21.4%) | 9 (16.7%) |  |  |
|  | Other determined etiology | 12 (2.1%) | 2 (3.7%) |  |  |
|  | Undetermined etiology | 142 (25.0%) | 12 (22.2%) |  |  |
| IIbIIIa receptor inhibitor (n, %) | | 266 (46.7%) | 21 (38.9%) | 1.23 | 0.268 |
| ASITN/SIR (n, %) | 0-1 | 248 (44.4%) | 23 (44.2%) | 0.39 | 0.825 |
|  | 2 | 207 (37.1%) | 21 (40.4%) |  |  |
|  | 3-4 | 103 (18.5%) | 8 (15.4%) |  |  |
| Onset to groin puncture time (median, Q1, Q3) | | 6.35 (4.15,11.43) | 5.48 (3.77,9.02) | -0.9 | 0.126 |
| Puncture to reperfusion time (median, Q1, Q3) | | 75.00 (55.00,110.00) | 82.00 (57.50,118.75) | -1.53 | 0.369 |
| mTICI 0-2a (n, %) | | 97 (17.3%) | 13 (24.1%) | 1.68 | 0.432 |
| 72h ICH (n, %) | | 169 (29.7%) | 22 (40.7%) | 2.83 | 0.093 |
| 72h sICH (n, %) | | 20 (3.5%) | 4 (7.4%) | 2.01 | 0.157 |

**Note**: Data are mean ± standard deviation, median (interquartile range), or frequency (percent).“*”indicates p＜0.05;**Abbreviations**: ASITN/SIR: American Society of Intervention Therapeutic Neuroradiology/Society of Interventional Radiology; ASPECTS Alberta Stroke Program Early CT Score；CONUT: controlling nutritional status score; ICH: intracranial haemorrhage;LDL: low density lipoprotein cholesterol. NIHSS: National Institutes of Health Stroke Scale; mTICI: modified thrombolysis in cerebral infarction,; PNI：prognostic nutritional index; TG: triglyceride; SBP：systolic blood pressure; sICH: symptomatic intracranial haemorrhage. TOAST: Trial of Org 10172 in Acute Stroke Treatment；WBC: white blood cell;

**Figure S1**


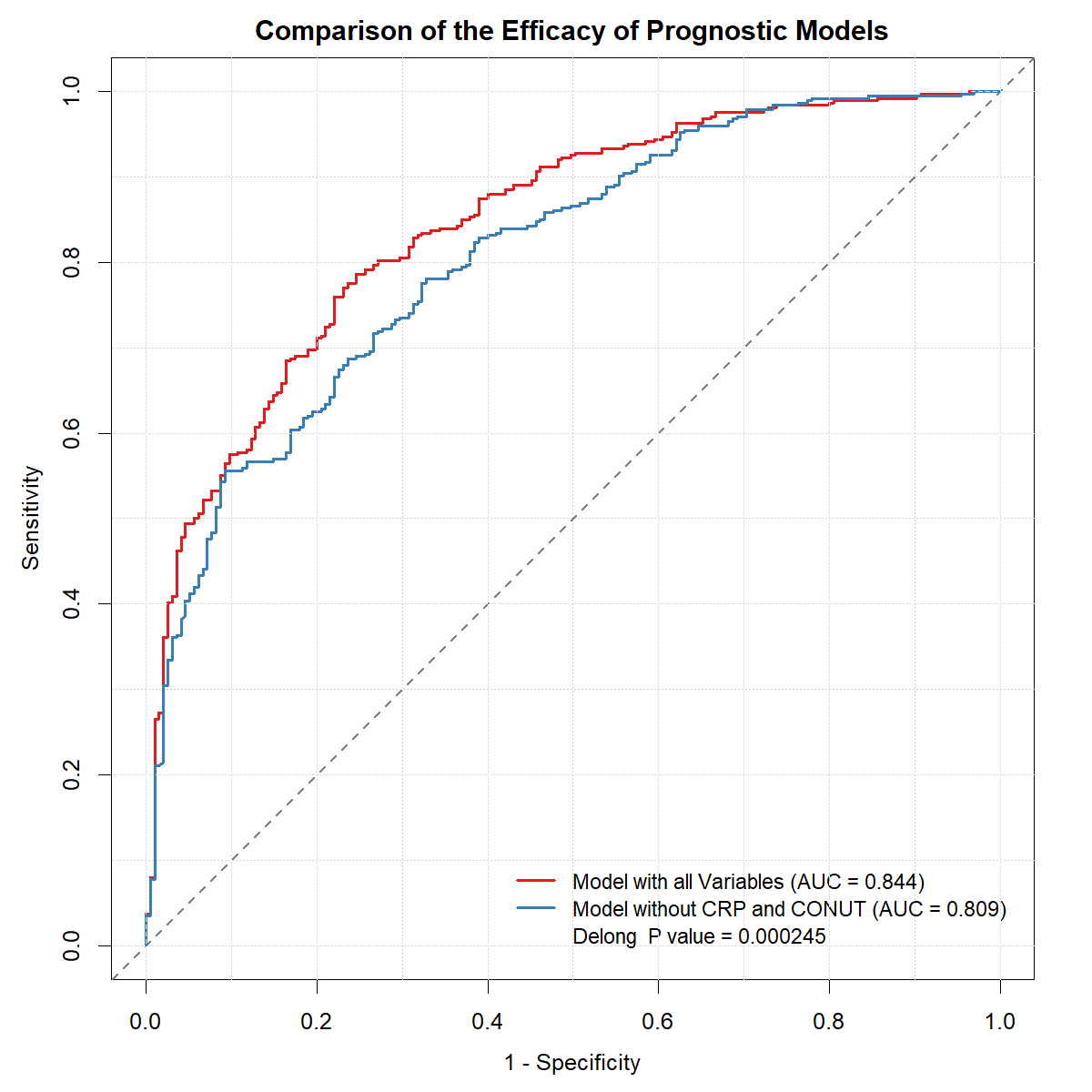


**Figure S1：Comparison of efficacy of different prognostic models.** Prognostic models were constructed based on logistic regression analysis. Model 1 (red curve) included all the significant variables: NIHSS + CRP + Puncture to reperfusion time + gender + Prior ischemic stroke/ TIA + mTICI+ ICH72H + CONUT, AUC=0.844; Model 2 (blue curve) included all the variants in Model 1 except CRP and CONUT, AUC=0.809. Delong test P value = 0.0002, meaning that CRP and malnutrition status contributes to better predicting performance. **Abreviations:** AUC: area under the curve; CI: confidence interval; CONUT: controlling nutritional status score; CRP: C reactive protein.
